# Microencapsulation of Curcumin in Crosslinked Jelly Fig Pectin Using Vacuum Spray Drying Technique for Effective Drug Delivery

**DOI:** 10.3390/polym13162583

**Published:** 2021-08-04

**Authors:** Nina Hartini, Thangavel Ponrasu, Jia-Jiuan Wu, Malinee Sriariyanun, Yu-Shen Cheng

**Affiliations:** 1 Department Chemical and Materials Engineering, National Yunlin University of Science and Technology, 123 University Road, Section 3, Douliu, Yunlin 64002, Taiwan; d10915002@yuntech.edu.tw (N.H.); tponrasu@gmail.com (T.P.); 2 Department of Nutrition, China Medical University, 91 Hsueh-Shih Rd., Taichung 40402, Taiwan; jjwu@mail.cmu.edu.tw; 3 The Sirindhorn International Thai-German Graduate School of Engineering, King Mongkut’s University of Technology North Bangkok, Bangsue, Bangkok 10800, Thailand; malinee.s@tggs.kmutnb.ac.th

**Keywords:** microencapsulation, curcumin, jelly fig pectin, vacuum spray drying, drug delivery

## Abstract

Microencapsulation of curcumin in jelly fig pectin was performed by the vacuum spray drying (VSD) technique. The VSD was advanced with a low inlet temperature of 80–90 °C and low pressure of 0.01 mPa. By the in situ cross-linking with multivalent calcium ions, jelly fig pectin produced stable curcumin encapsulated microparticles. The physiochemical characteristics of microparticles were thoroughly investigated. The results revealed that 0.75 *w*/*w*% of jelly fig pectin and inlet temperature of 90 °C could be feasible for obtaining curcumin microparticles. The VSD technique showed the best encapsulation efficiency and yield and loading efficiency was up to 91.56 ± 0.80%, 70.02 ± 1.96%, and 5.45 ± 0.14%, respectively. The curcumin was readily released into simulated gastrointestinal fluid with 95.34 ± 0.78% cumulative release in 24 h. The antioxidant activity was stable after being stored for six months and stored as a solution for seven days at room temperature before analysis. Hence, the VSD technique could be applicable for the microencapsulation of bioactive compounds such as curcumin to protect and use in the food/pharmaceutical industry.

## 1. Introduction

Several techniques have been developed in recent years to improve the applications of bioactive compounds in the food, pharmaceutical, and cosmetic sectors. Despite many methods available to protect the bioactive compounds, microencapsulation technology has emerged as one of the most attractive methods to safeguard bioactive compounds such as vitamins, essential oils, volatile chemicals, pigments, and more [1]. Microencapsulation is a process for producing and protecting bioactive compounds from the external environment, including light, oxygen, and temperature, by suitable matrices to improve their stability, taste, flavor, and color [2,3,4]. In the health sector, microencapsulation is mainly used for the sustained release of drugs [5]. Microencapsulation can be accomplished by many different measures, such as coacervation, extrusion, and spray drying [6,7,8].

**Figure 1:**
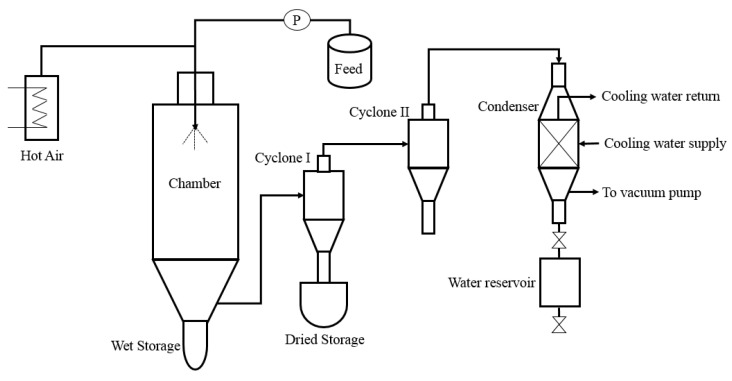
Schematic diagram of vacuum spray drying method.

Vacuum spray drying (VSD) represents one of the new drying strategies for microencapsulation, which can be used to preserve the flavor and structure of bioactive molecules [9]. The principle of VSD resembles conventional spray drying, where the emulsion is spraying into the drying chamber with the help of an atomizer. Then, the atomized droplets brought into contact with the hot air simultaneously produced granules in the chamber through rapid moisture evaporation of the solvent (Figure 1) [10]. The VSD was operated at low temperature and pressure in order to avoid the damage of the bioactive compounds [11]. The VSD was used to produce dried probiotic powder to develop probiotic foods [12]. The VSD method has a high reproducible capacity with a more diverse matrix, resulting in high encapsulation efficiency [13].

Curcumin (1,7-bis (4-hydroxy-3-methoxyphenyl)-1,6-hepta-dien-3,5-dione) is a natural pigment polyphenol extracted from turmeric plant (*Curcuma longa*) [14]. Curcumin has several functions: antioxidant, antitumor, anticarcinogenic, and anti-inflammatory [15,16]. In clinical studies, curcumin is observed to have many benefits and is safe to use at high doses. However, its physical characteristics limited its use in oral pharmaceutical delivery due to very low solubility. A curcumin compound is susceptible to light, temperature, moisture, and oxygen. Curcumin is less stable at acidic pH levels and it decomposes rapidly at extreme pH levels [16,17].

Many different polymeric matrices had been evaluated for encapsulation of curcumin [18]. Ideally, a polymeric matrix should be biocompatible, biodegradable, non-toxic, water-soluble, and inexpensive [14]. Pectin is an abundant natural biopolymer composed of straight-chain molecules of α (1,4)-linked ᴅ-galacturonic acid that can be extracted from various sources and can be employed as a matrix for the microencapsulation [19]. Nevertheless, most pectin presented in the fruit peels are high methoxyl pectin and their isolation requires using elevated temperature and chemical extraction process, which generates acidic wastewater that can cause environmental problems [20]. The jelly fig (Ai-Yu) is derived from the Moraceae family growing in the central mountains of Taiwan. The aqueous extract of jelly fig achenes is primarily composed of pectinous polysaccharides [21] with other compounds including pectinesterase, chitinase, protein, and pectinesterase inhibitor and exhibits antibacterial antioxidant properties [22]. Unlike the high methoxy pectin extracted from fruit peels that required harsh extraction conditions, the jelly fig pectin is low methoxy pectin that can be easily extracted by water at room or mild temperature. Low methoxy pectin can form a gel in the presence of multivalent cations under acidic condition. The gelation is established through “egg-box” binding structure because of the formation of intermolecular junctions between pectin chains [3]. It is possible to increase trapping effectiveness of the core material by stirring it into a crosslinked anionic polymer with calcium ions (Ca^2+^) [23,24]. In addition, the pectin yield of jelly fig is around 15% [19], which is also higher than its close species *Ficus pumila Linn.* that can be found commonly in East Asia [19]. Therefore, the jelly fig pectin could be a new source of low methoxy pectin for microencapsulation and sustained drug release [25].

In this study, curcumin was used as a model lipophilic microencapsulated in a polysaccharide matrix (jelly fig pectin) using vacuum spray drying technology. In the longer term, jelly fig pectin-based formulations can be developed to enhance curcumin dispersion, stability, and sustained release. As a result, the encapsulated curcumin is expected to be more bioavailable and effective in human beings. Therefore, the aim of this study is to investigate the potential of jelly fig pectin as an encapsulant for the production of curcumin microcapsules by in situ crosslinking with calcium ions by using the alternative drying method VSD at low temperatures and low pressures. The physicochemical properties and storage stability of the prepared microcapsules were thoroughly characterized and assessed. The in vitro release of curcumin was also observed in the simulated gastrointestinal fluids.

## 2. Materials and Methods

### 2.1. Materials

Jelly Fig achenes and corn oil were bought from the local stores. Curcumin (*Curcuma longa* ≥ 65% purity), Polysorbate 80, dicalcium phosphate hydroxide, sodium citrate dihydrate, hydrogen chloride, ethylenediaminetetraacetic acid (EDTA), and β-galactoside were purchased from Sigma-Aldrich Co., Ltd. (St. Louis, MO, USA). Succinic acid, 2,2-Diphenyl-1-picrylhydrazyl (DPPH), and potassium bromide were obtained from Alfa Aesar (Ward Hill, MA, USA). All chemicals used in this work were of reagent grade, if not particularly specified.

### 2.2. Methods

#### 2.2.1. Extraction of Pectin from Jelly Fig Achenes

The extraction of pectin was carried out as in our previous study with slight modifications [19]. Briefly, 10 g of jelly fig achenes were put in 400 mL of aqueous solution (ratio 1:40) containing 0.1 M EDTA and the pH was adjusted to pH 8 using 4 M NaOH. Extraction was performed at 240 rpm for 2 h at 70 °C. The jelly fig achenes were then removed by filtering through a cheese cloth. Three volumes of ethanol were added to the filtrate to precipitate the jelly fig pectin at room temperature for 2 h. Then, the precipitate was dried in an oven (model DOS60, Deng YNG, Taiwan) at 45 °C for 2 days followed by pulverizing using a knife mill. The jelly fig pectin powder was stored at room temperature until further use.

#### 2.2.2. Curcumin-Jelly Fig Pectin Microcapsules (C-JFMs) Production

The preparation of microcapsules was achieved using the previous method with slight modification [3]. Briefly, the 100 mL aqueous core solution composed of 0.5 *w*/*w*% curcumin, 0.75 *w*/*w*% corn oil, 3.75 *w*/*w*% Tween 80, 0.6 *w*/*w*% succinic acid, and pH adjusted to 5.6 with ammonium hydroxide. A matrix solution composed of 1.5–4.5 *w*/*w*% jelly fig extract, 0.075 *w*/*w*% dicalcium phosphate hydroxide, and 0.018 *w*/*w*% sodium citrate dehydrate in 100 mL aqueous solution. Then, these two solutions were homogenized in the ratio of 1:1 at 9500 rpm for 2 min with a hand-held homogenizer (Model D9KC-CK100, Dogger, Taiwan). The size of the emulsion was scaled down using Sonicator (Linko Ultrasonic Processor by Hanchen, Luyi, China) at power 95%, on/off: 10 s/10 s for 2 min. Then, the mixture was directly pumped into a YC-510 Lab Spray Dryer to produce microcapsules. The conditions used were 80 °C, 90 °C of inlet temperature, the pressure of 0.01 mPa, and 5 rpm of feed peristaltic pump, respectively. The microcapsules obtained were stored in a desiccator until further use.

#### 2.2.3. Size Distribution and Viscosity of the Emulsion

The particle size distributions and polydispersity index (PDI) of emulsion were measured using a dynamic light scattering (DLS) by a Zeta Plus Analyzer (Brookhaven Instruments Corporation, Holtsville, NY, USA) before VSD. Each sample was diluted 20× and was measured independently in triplicate [3]. The viscosity was determined by Vibro viscometer SV-10/SV-100 (A&D, Tokyo, Japan). The viscometer was calibrated using a standard viscosity fluid with the known value of viscosity and density of 0.93 mPa.s at 23 °C using DI water. The sample was added to the disposable cup until 10 mL, then measured for 10 min.

#### 2.2.4. Yield and Moisture Content of C-JFMs

The yield was calculated by comparing the weight of microcapsules produced against the total weight of the microcapsules forming material using Equation (1) [26]. The moisture content was also determined thermogravimetrically by drying the samples in an oven for 24 h at 105 °C using Equation (2) [27].

(1) $$\mathrm{Yield}\;(\%)=\frac{\mathrm{Weight}\;\mathrm{of}\;\mathrm{microcapsules}}{\mathrm{Total}\;\mathrm{weight}\;\mathrm{initial}}\;\text{×}\;100\%$$

(2) $$\%\;\mathrm{Moisture}\;\mathrm{Content}=\frac{\mathrm{weight}\;\mathrm{of}\;\mathrm{plate}\;-\mathrm{weight}\;\mathrm{total}\;\mathrm{before}\;\mathrm{drying}}{\mathrm{weight}\;\mathrm{total}\;\mathrm{before}\;\mathrm{drying}-\mathrm{weight}\;\mathrm{total}\;\mathrm{after}\;\mathrm{drying}}\;\;\text{×}\;100\%$$

#### 2.2.5. Drug Loading Efficiency (LE) and Encapsulation Efficiency (EE)

The drug loading and encapsulation efficiency were determined using Equations (3) and (4) according the previous method [15]. Briefly, the total curcumin content was calculated by dissolving 25 mg of C-FMs in 25 mL 60% ethanol solution. Then, the solution was centrifuged at 5000 rcf for 5 min and the concentration of free curcumin in the supernatant was determined. All the samples used were triplicate and their absorbances at 425 nm were measured using a UV-Vis spectrophotometer.

(3) $$\mathrm{Drug}\;\mathrm{loading}\;\mathrm{efficiency}=\frac{\mathrm{Total}\;\mathrm{curcumin}-\mathrm{Free}\;\mathrm{curcumin}}{\mathrm{Mass}\;\mathrm{of}\;\mathrm{Microcapsules}}\;\text{×}\;100\%$$

(4) $$\mathrm{Encapsulation}\;\mathrm{efficiency}=\frac{\mathrm{Total}\;\mathrm{curcumin}-\mathrm{Free}\;\mathrm{curcumin}}{\mathrm{Total}\;\mathrm{curcumin}}\;\text{×}\;100\%$$

#### 2.2.6. The Characterization of C-JFMs

The morphology, shape, and size of the C-JFMs were analyzed by Scanning Electron Microscopy (SEM). The samples were analyzed at 15 kV with gold/palladium coating (JOEL Ltd., model JSM-6701F, Peabody, MA, USA). The particle size measurement was performed using ImageJ Software (Version 1.50i). The functional groups of the samples were analyzed by using a Fourier Transform Infrared Spectrometer (FTIR) (Perkin Elmer, Inc., Waltham, MA, USA). The samples were prepared as KBr pellets and the infrared spectra were collected with 16 scans from the wavenumber of 450–4000 cm^−1^ with a resolution of 8 cm^−1^. The thermal stability of the material was analyzed using thermogravimetric analysis (Perkin Elmer, Inc., Waltham, MA, USA) from 30–600 °C with a heating rate of 10 °C/min and nitrogen flow of 20 mL/min [14].

#### 2.2.7. Antioxidant Stability during Storage

The stability of antioxidant activity was evaluated by using the DPPH method and calculated using Equation (5) [28]. Briefly, the 1 mL of sample (C-JFMs) was mixed slowly with 2 mL of 0.2 mM DPPH solution and incubated in the ambient temperature and in the dark for 30 min. The absorbance was measured at 517 nm using a UV-Vis spectrophotometer. The solution without a sample was used as a control. The study was performed in triplicate.

(5) $$\mathrm{Antioxidant}\;\mathrm{activity}=\frac{\mathrm{The}\;\mathrm{absorbance}\;\mathrm{of}\;\mathrm{control}-\mathrm{Absorbance}\;\mathrm{of}\;\mathrm{sample}}{\mathrm{Absorbance}\;\mathrm{of}\;\mathrm{control}\;}\;\text{×}\;100\%$$

#### 2.2.8. In Vitro Release Study

Curcumin release was observed using 25 mg C-JFMs incubated in Simulated biological fluids: simulated gastric fluid (SGF prepared wiht 34.2 mM NaCl in DI water and adjusted to pH 1.2 by 4 M HCl), simulated intestinal fluid (SIF prepared with 50 mM KH_2_PO_4_ and adjusted to pH 6.8 by 4M NaOH), and Simulated Colonic Fluid (SCF prepared with 50 mM KH_2_PO_4_, 0.2% Tween and 0.13 U/mL β-galactosidase and adjusted to pH 7.4 by 4M NaOH). First, the sample was immersed in 25 mL SGF for 2 h. Then, the microcapsules were transferred into 25 mL SIF and incubated for 4 h. Finally, the microcapsules were transferred into SCF and incubated for 18 h. The suspension was continuously stirred at 100 rpm, 37 °C during the release study. The release medium was centrifuged at 5000 rcf for 5 min. Then, the supernatant was measured at 425 nm using a UV-Vis spectrophotometer. The pellet of the SGF was sequentially used for SIF and SCF analysis [3,29,30].

#### 2.2.9. Statistical Analysis

All the experiment was performed in triplicate and the data were used for statistical analysis. The significant difference was evaluated using One-way ANOVA by Design Expert Software version 11.1.0.1. The level of significance was considered as *p* < 0.05.

## 3. Results and Discussion

### 3.1. Particle Size Distribution and Viscosity of the Emulsion

**Figure 2:**
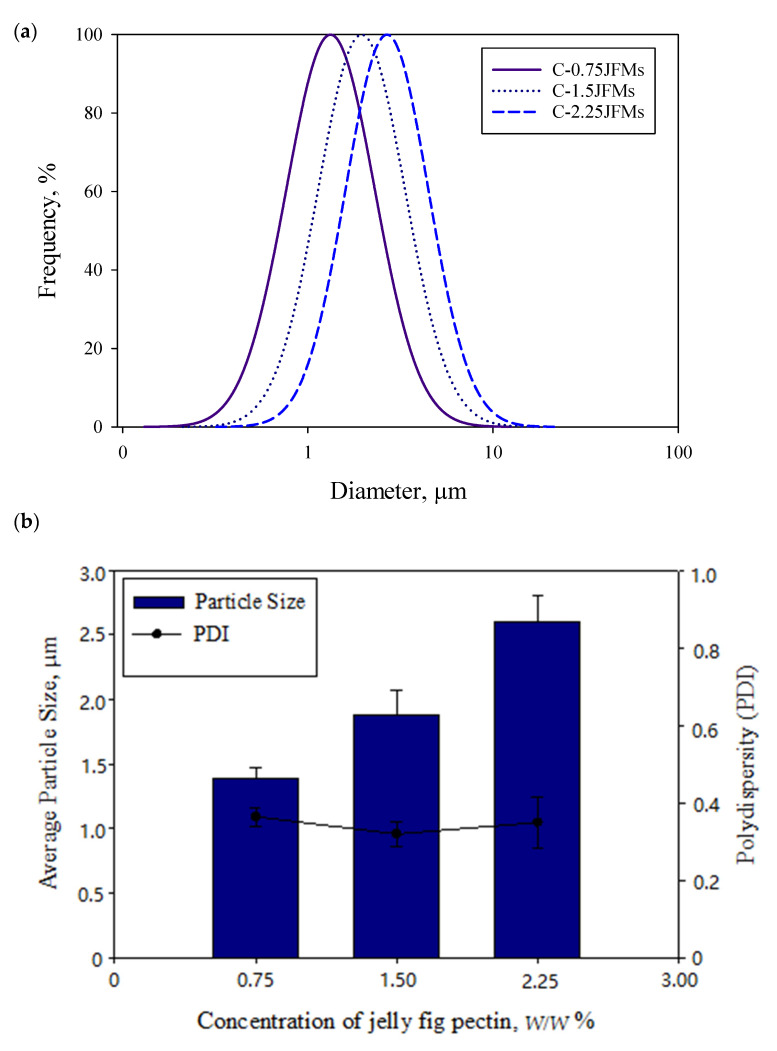
The size distribution of the emulsion, (**a**) the average diameter, and (**b**) polydispersity index before VSD.

The emulsion properties are crucial in the success of the microencapsulation process. The most important properties of the emulsion before the VSD process are the droplet size, polydispersity, and viscosity [31]. By emulsifying, jelly fig pectin increased the viscosity of the emulsion, resulting in a larger average diameter of droplets [32]. The larger droplet size and higher viscosity of the emulsion could interfere with the atomization process by narrowing the size distribution [3]. In addition, high viscosity increases the internal resistance of droplets, which could affect the outcome of the atomization process. The average particle size and PDI of the prepared emulsions were displayed in Figure 2a,b. The formulation C-0.75JFMs, C-1.5JFMs, and C-2.25JFMs had particle sizes of 1.39 ± 0.86 µm, 1.89 ± 0.19 µm, and 2.61 ± 0.20 µm and the corresponding PDI of 0.36 ± 0.02, 0.32 ± 0.03, and 0.35 ± 0.07, respectively. The PDI represents the distribution of uniform particles in a sample; the more small the PDI represents, the more uniform sample [3]. In a published report, the in situ cross-linked alginate microcapsules of corn oil showed the average droplet size in the range of 8.1 ± 0.2 to 17.9 ± 0.3 µm [3]. The microencapsulation of grapeseed oil emulsion prepared with gum Arabic and gum Arabic/Maltodextrin also demonstrated the particle size of 6.47 ± 0.06 µm and 5.80 ± 0.11 µm, respectively [33].

**Figure 3:**
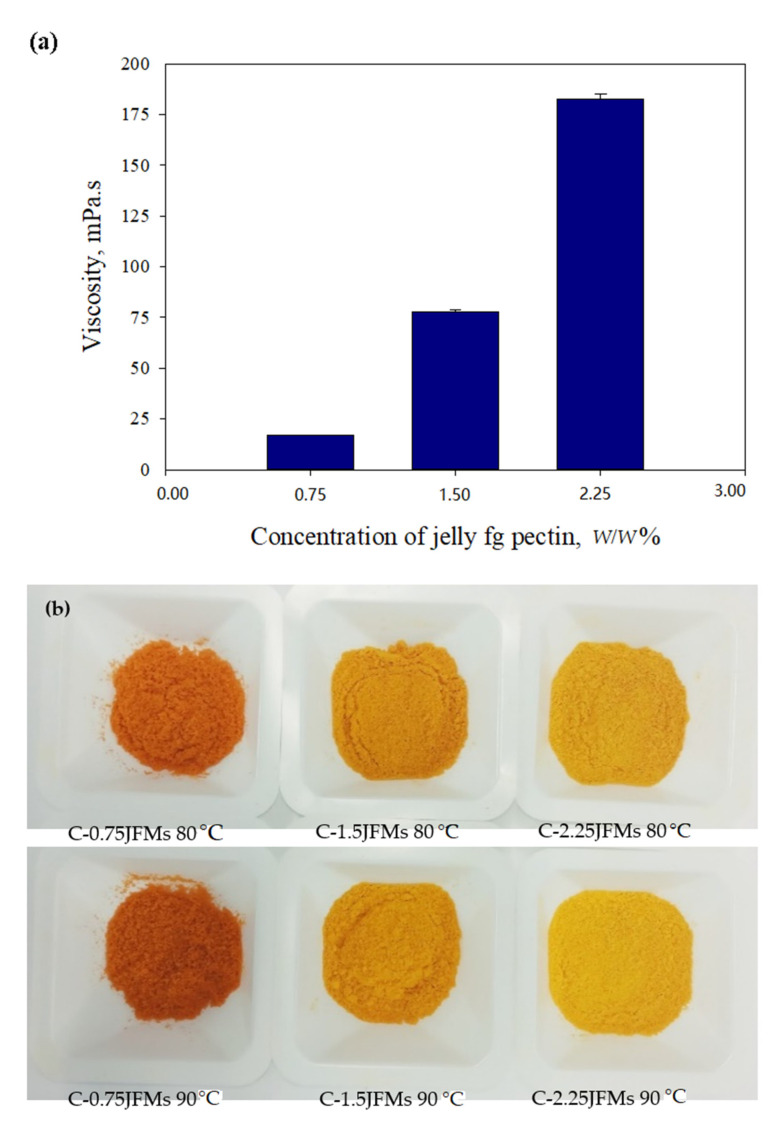
The viscosity of emulsion (**a**) before VSD and (**b**) dried powder of C-JFMs.

The emulsions prepared with jelly fig pectin as matrix exhibited low viscosity of the range from 17.02 ± 5.61 to 186.47 ± 25.03 mPa.s) (Figure 3a). The viscosity value of the emulsion pumped into the VSD depends on the emulsifying ability, water solubility, and concentration of the matrix. The low viscosity of the emulsion could generally produce small size droplets of atomization [32]. Therefore, the matrix with low viscosity, such as jelly fig pectin, could be useful for the effective release of microcapsules [34].

### 3.2. The Yield and Moisture Content of Microcapsules

The electrostatic interaction by ion gelation created a stable matrix for curcumin to be easily encapsulated and released from the matrix in the gastrointestinal fluids [2,3,14]. The results (Table 1) indicated that the higher concentration of jelly fig pectin could produce microcapsules with a higher mass quantity. The total dried microcapsules of C-JFMs were around 4.63 ± 0.02 to 6.32 ± 0.92 g. The appearance of dry microcapsules was orange-yellow, illustrating that some free curcumin presented on the surface of the matrix. The highest yield of microcapsules was around 70% obtained from the formulation of C-0.75JFMs 90. The results were similar to the data shown in other reports in which the overall yield of spray drying on a laboratory scale was limited and found around to be 50–70% [35]. The low yields of microcapsules could occur due to product loss during drying and sample harvest processes. During the VSD, product loss was also observed due to stuck-in chambers, tubes, cyclones, and collecting vessels.

The moisture content was also an essential parameter for the quality of encapsulation. Generally, the quality of microcapsules depends on the nature of dried powder and granules with low moisture content [31]. The low moisture content limits the water-absorbing capacity of the encapsulant and it is better to avoid microbial growth that could cause severe damage to the product [36]. In addition, the moisture content of microcapsules also affects the texture, appearance, stability, and shelf-life. The moisture contents of microcapsules obtained in this study were all below 6.6%, which is slightly higher than some microcapsules prepared using different matrices but still at the level of low moisture content [37,38].

### 3.3. Drug Loading Efficiency (LE) and Encapsulation Efficiency (EE)

The efficiency of encapsulation was influenced by several factors, including solubility of a matrix in organic solvents, the solubility of organic solvent in water, concentration of matrix, the ratio of dispersed phases to continuous phases, and the evaporation rate of solvents [39]. The suitable formulation of the matrix and optimum conditions can produce proper microcapsules with desired characteristics. The encapsulation efficiency obtained in study was in the range of 68.95 ± 1.14 to 91.56 ± 0.80%. The highest encapsulation efficiency was obtained from the formulation C-2.25JFMs 90. The drug loading efficiency of curcumin was measured based on the weight of curcumin that can be accommodated by each total microcapsule and the results were showed in Table 2.

The results were in line with the earlier study in which fish oil encapsulated whey protein matrix produced an encapsulation efficiency of 57.59 to 70.20% by spray drying [40]. The encapsulation efficiencies of 63.39 to 80.97% were also noticed for flurbiprofen encapsulated in chitosan by spray drying [41]. Hence, the loading efficiency measured the weight of curcumin present in microcapsules. It was reported that there were less free drugs at the surface of the microcapsules, showing the higher drug loading efficiency [42].

The purpose of evaluating the encapsulation efficiency in microcapsules was to determine the ability of the matrix and inlet temperature VSD during the absorption of the curcumin. The innovation of microencapsulation by VSD method in low temperature using jelly fig pectin as a matrix by in situ crosslinking increased the encapsulation efficiency. The results confirmed that VSD could be a feasible process for improving the quality of polyphenols sensitive to high-temperature [28]. Moreover, calcium ions interact with the carboxylic groups in the jelly fig pectin and, hence, the polymer chains of pectin became dense, resulting in a strong matrix in the system [43].

### 3.4. Morphology and Particle Size Distribution

**Figure 4:**
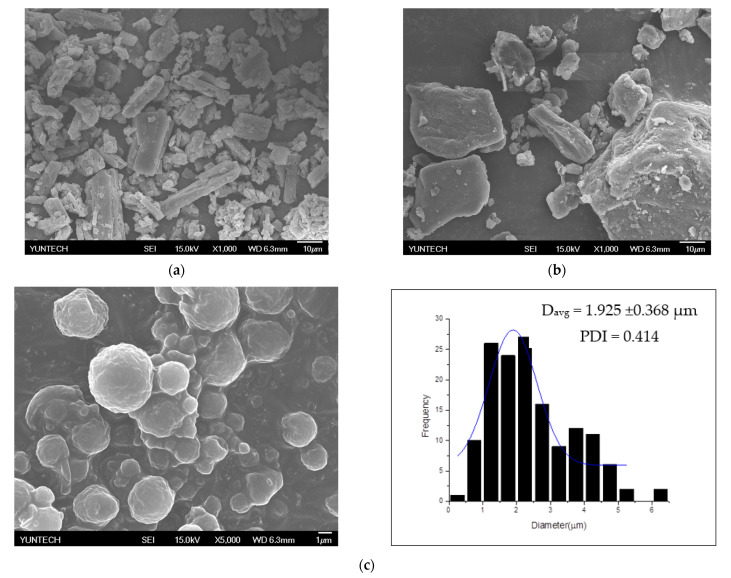

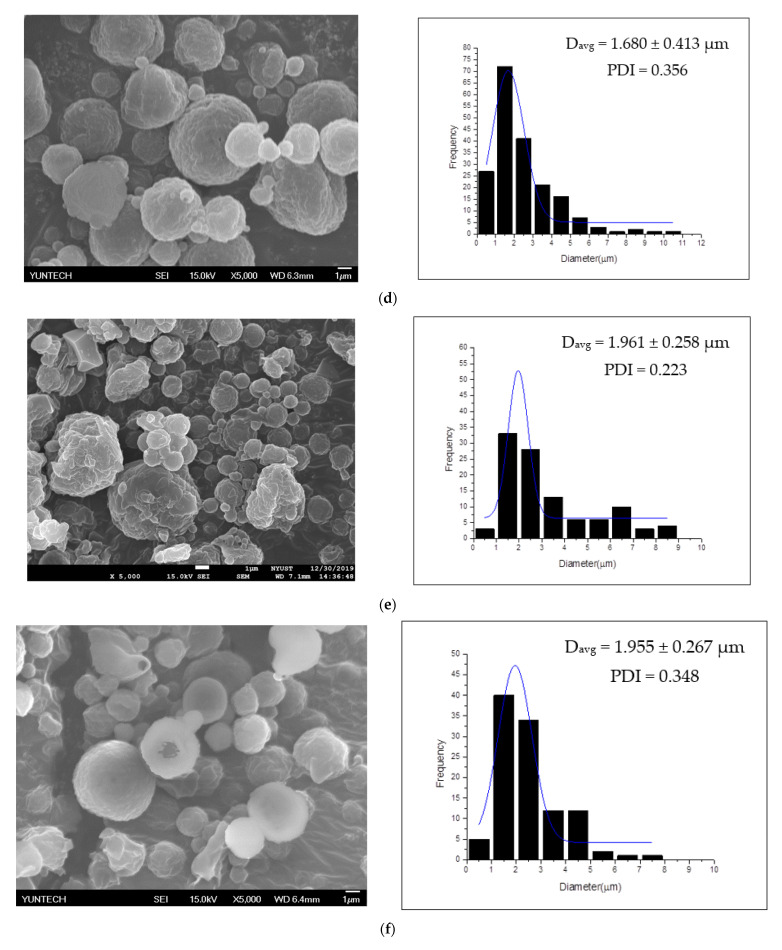

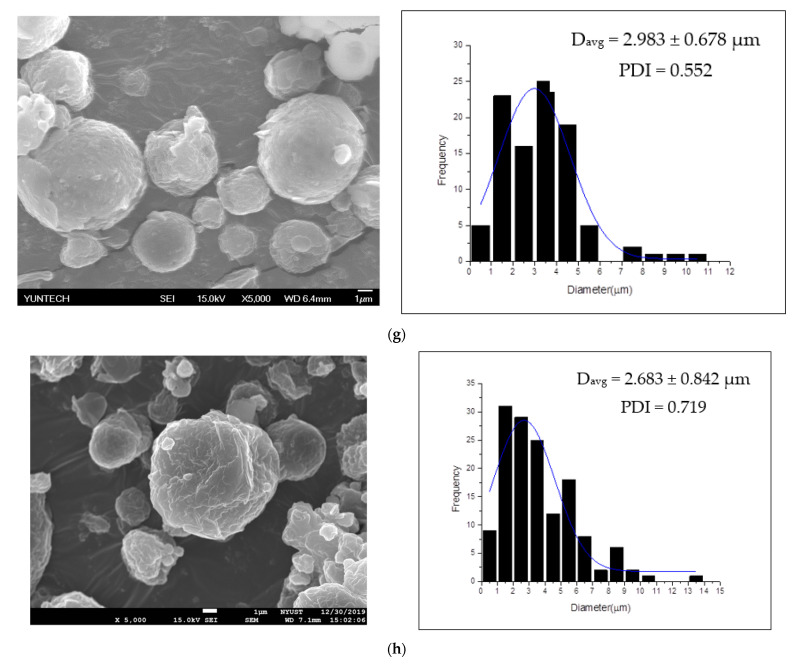
Morphology of (**a**) curcumin (**b**) extract of jelly fig at 1000×, (**c**) Morphology and particle size distributions of C-0.75JFMs 80 °C, (**d**) C-1.5FJMs 80 °C, (**e**) C-2.25JFMs 80 °C, (**f**) C-0.75JFMs 90 °C, (**g**) C-1.5FJMs 90 °C, and (**h**) C-2.25JFMs 90 °C.

The morphology of curcumin and jelly fig pectin was observed in SEM images (Figure 4a,b). Results indicated that the original particle of curcumin and jelly fig pectin microcapsules resembled crystal and were irregular in shape. The external morphology of curcumin microcapsules was shown in Figure 4c–h. The particle size and morphology of the particles obtained through VSD were round and intact (irregular). The encapsulation process showed excellent physical integrity, such as being free of cracks observed from the external morphology of the encapsulants [44]. The microstructure of the round shape indicated that the absorbed water in the matrix did not evaporate by contact with hot air. A smooth round surface morphology indicated that the microcapsules were stable between the solvent, the wall material, and the core [45]. The dented microstructure inferred that the particle shrinkage might have occurred during the VSD process [32].

The high-pressure homogenization and rapid drying produced smaller particles and morphologies without shrinking [13]. The increased particle size generally resulted in a slower diffusion rate and molecules required more time to reach the surface atomization of the wall material during the drying process [46]. Figure 4c–h showed that the average diameter of microcapsules ranged from 1.68 ± 0.41 to 2.98 ± 0.68 µm with a PDI around 0.22 to 0.72. The average particle size of taro starch and almond oil increased after spray drying showed a wide range of particle sizes from 1.6 to 31.1 µm [1]. As a result, we also found that the size of curcumin microcapsules in jelly fig pectin is smaller than in Gum Arabic and Gum Arabic/maltodextrin. Gum Arabic and Gum Arabic/Maltodextrin showed average droplets of 5.80 ± 0.11 to 6.47 ± 0.06 µm and, after spray drying, it was around 20 to 30 µm [33].

### 3.5. Fourier Transform Infrared (FTIR) Spectroscopy

**Figure 5:**
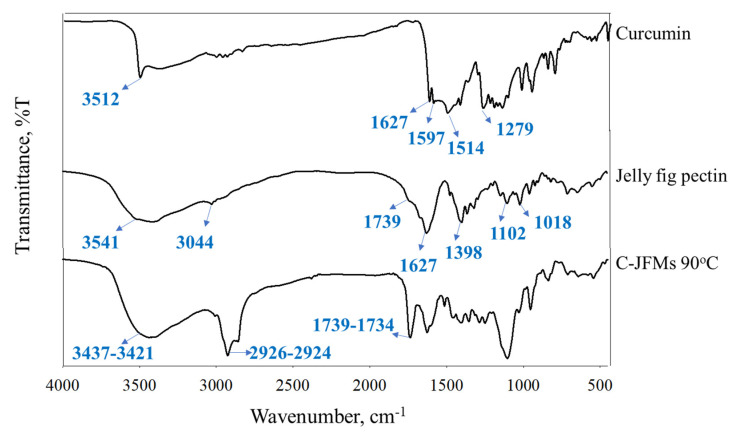
FTIR analysis of curcumin, jelly fig pectin, and microcapsules.

FTIR was carried out to determine the characterization and potential biomolecular conjugation of microcapsules and showed in Figure 5. A peak at 1627 cm^−1^ attributed predominantly to the overlapping stretching of aromatic (C=C) at 1597 cm^−1^ of the benzene ring. The curcumin ligand showed stretching vibrations of phenolic O-H at 3512 cm^−1^ and C=C and C=O at 1514 cm^−1^. Furthermore, a significant intense band was observed between 1279 and 1116 cm^−1^ and attributed to the bending vibration of the (C–O) aromatic group [47].

The main characteristic peaks of the jelly fig pectin (pectin type LM) observed were provided in Figure 5. The peak was observed at 3541 cm^−1^ and indicated the OH stretching vibration and bending of low methoxyl pectin. The peaks observed at 3044 cm^−1^ and 1739 cm^−1^ were attributed to the C–H stretching of epoxide ring and carbonyl (C=O) group. The peaks noticed at 1627 cm^−1^ and 1398 cm^−1^ denoted the axial deformation of the C–C double bond. The peaks located at 1102 cm^−1^ and 1018 cm^−1^ were attributed to the bending vibration of the (C–O) phenolic band and CH–O–CH stretching vibration, respectively [48].

The FTIR spectra of C-JFMs showed a strong peak between 3437–3421 cm^−1^ attributed to OH stretching and the hydrogen bridges of microcapsules by calcium ions (Figure S1) [49]. The strong peak exists between 2926–2924 cm^−1^ and 1739–1734 cm^−1^ confirmed the presence of asymmetric C–H stretching, symmetric–CH stretching, and carbonyl group [29]. The broader peak indicated a strong interaction between jelly fig pectin with calcium ion due to a chemical reaction between an acid (COOH) and a basic (OH) group [50]. Furthermore, the characteristic peaks of pure curcumin were also observed in the physical mixture of microcapsules [51].

### 3.6. Thermogravimetric Analysis (TGA)

**Figure 6:**
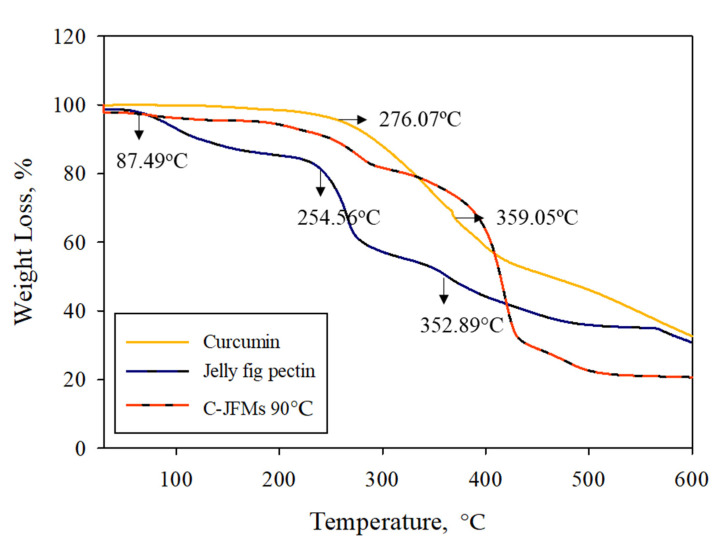
Thermogravimetric curves for curcumin, jelly fig, and microcapsules.

The TGA analysis displayed the intermolecular interactions of the microcapsules under high-temperature in terms of thermal stability of the vacuum spray-dried microcapsules (Figure 6) [14]. The curcumin thermogram did not show any water loss due to its hydrophobic nature [50]. The curcumin started to decompose at 276.07 °C and the maximum degradation temperature was observed at 359.05 °C with a mass loss of 28.83% and the final residue of 32.66%. Jelly fig pectin showed three stages of weight loss: the first for a dehumidification process at 87.49 °C, the second peak for the evaporation of internally bound water in the polymer structure at 254.56 °C, and the third at 352.89 °C for polymer decomposition. The crosslinking of jelly fig pectin with calcium ions increased the thermal stability of microcapsules (Figure S2). Curcumin showed higher thermal stability compared to jelly fig pectin. Therefore, the results concluded that the formation of the polymer network considerably improved the physical properties and thermal stability of jelly fig pectin in the microcapsules [52]. In order to maintain their structural integrity for their final use and during manufacturing, microcapsules need to have good thermal characteristics and mechanical strength [53].

### 3.7. Antioxidant Stability during Storage

**Figure 7:**
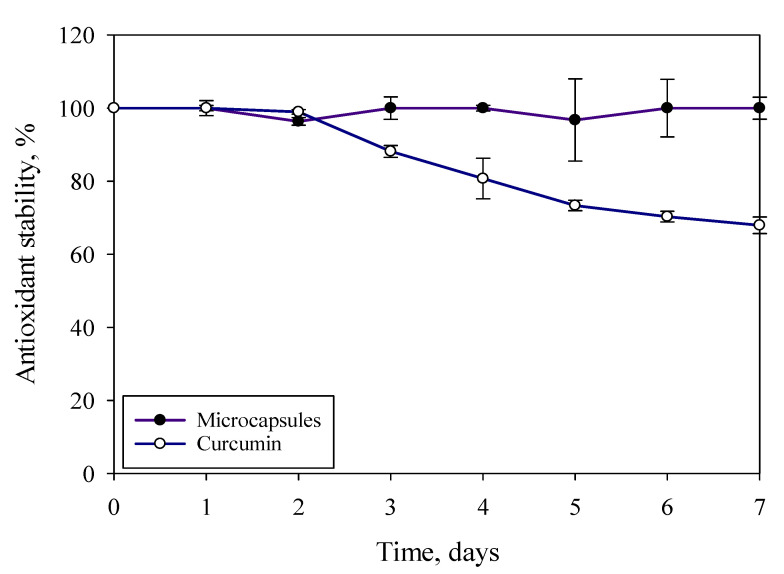
Antioxidant stability of curcumin and microcapsules after being stored in a desiccator for 6 months and antioxidant stability for 7 days in the room temperature.

Previous studies have shown that curcumin is highly susceptible to degradation by light, pH, and temperature [54]. The microcapsules prepared from the formulation C-JFMs were initially stored at room temperature for six months and then immersed in the buffer in order to analyze antioxidant stability. The antioxidant stability analysis was conducted every day for seven days. When compared with pure curcumin, the microcapsule had relatively stable storage stability and did not exhibit significant degraded antioxidant activity during storage (Figure 7). This is mainly due to the crosslinking of jelly fig pectin which used calcium ions to protect the flavor of curcumin and protected antioxidant activity. Moreover, the OH phenolic group and methylene CH_2_ group of jelly fig pectin were responsible for increasing its antioxidant activity [51].

### 3.8. In Vitro Release Studies

The controlled release of curcumin was observed by a diffusion study. The diffusion rate is primarily affected by permeability, solubility, chemical properties, morphology, and glass transition temperature of the wall material [34]. The low viscosity of the matrix and the ratio of the crosslinking agent are also crucial for drug release. The microencapsulation process using the VSD technique controlled the drug release and extended the shelf life of the encapsulated product. The jelly fig pectin was used as a hydrophilic matrix to release active ingredients [21]. Furthermore, the rate of release was activated slowly by enzymatic degradation and temperature of the release medium [55]. Curcumin can easily be oxidized and decomposed; therefore, the microencapsulation process was used to protect its chemical properties.

**Figure 8:**
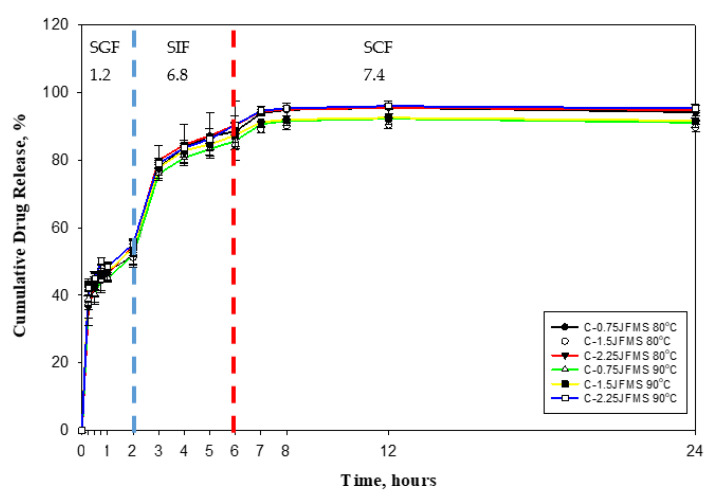
The cumulative drug release profile.

The cumulative drug release of curcumin was shown in Figure 8. The initial curcumin releases observed from all formulations were in the range of 51.10 ± 2.27 to 55.23 ± 1.06% after two hours of incubation in SGF. Then, the cumulative release of curcumin slowly increased to a range of 84.35 ± 4.29 to 90.46 ± 2.61% after eight hours of incubation in SIF and, finally, 89.64 ± 1.11 to 95.34 ± 0.78% releases were noticed at the end (24 h) of incubation in SCF. The formulation C-2.25JFMs 90 showed a higher curcumin release due to the higher concentration of matrix might influence the release of curcumin [25]. The results concluded that C-JFMs could be suitable for drug delivery in the colonic region.

The cumulative release of curcumin in SGF was slightly higher than SIF and SCF and this is possibly due to the dissolution of free curcumin [51]. Moreover, the deionization of the carboxyl group residues of jelly fig pectin decreased the swelling ability of the polymer and drug release [25]. The protonated carboxyl group of jelly fig pectin in SGF at pH 1.2 forms basic conjugates through shrunken and aggregated microcapsules. Due to the ionization of carboxyl groups, the swelling ratio increases with increasing pH, which is caused by the repulsion of carboxyl groups within the pectin chains [30].

## 4. Conclusions

The microencapsulation of curcumin in jelly fig pectin by in situ crosslinking was successfully developed by using the VSD technique. The results showed that the best encapsulation efficiency, yield, and loading efficiency were 91.56%, 70.02%, and 5.45%, respectively. The developed microencapsulation technology improved the antioxidant stability of curcumin during storage. The cumulative release of curcumin in SGF and SIF was 95.34 ± 0.78% in 24 h. The characterization of the microcapsules produced from the formulation C-JFMs showed uniform morphology and particle size distributions. The results derived from this study recommended the VSD process could potentially be applied to encapsulate the curcumin and other lipophilic bioactives for the effective delivery and applications in the food or pharmaceutical industry.

## Figures and Tables

**Table 1:** Properties and data analysis of microcapsules.

Formulation	Dried Powder, g	Yield, *w*/*w*%	Moisture Content, *w*/*w*%
C-0.75JFMs 80 °C	4.63 ± 0.02	64.02 ± 0.17	6.64 ± 0.82
C-0.75JFMs 90 °C	5.07 ± 0.15	70.02 ± 1.96	5.51 ± 0.89
C-1.5JFMs 80 °C	5.53 ± 0.06	63.09 ± 1.00	6.44 ± 0.94
C-1.5JFMs 90 °C	5.62 ± 0.02	64.09 ± 0.30	6.36 ± 1.80
C-2.25JFMs 80 °C	5.72 ± 1.71	56.04 ± 6.78	6.16 ± 2.53
C-2.25JFMs 90 °C	6.32 ± 0.93	62.00 ± 9.10	3.84 ± 2.93

**Table 2:** Properties and analysis of drug loading efficiency and encapsulation efficiency.

Formulation	Total Curcumin, µg/mg	Free Curcumin, µg/mg	LE, %	EE, %
C-0.75FMs 80 °C	58.14 ± 0.58	18.03 ± 0.69	4.01 ± 0.07	68.95 ± 1.14
C-0.75FMs 90 °C	64.07 ± 1.36	9.80 ± 0.33	5.45 ± 0.14	84.77 ± 0.98
C-1.5FMs 80 °C	53.44 ± 1.29	10.71 ± 0.36	4.27 ± 0.10	79.95 ± 0.39
C-1.5FMs 90 °C	55.86 ± 1.80	6.59 ± 0.12	4.90 ± 0.17	88.19 ± 0.22
C-2.25FMs 80 °C	48.23 ± 1.29	4.24 ± 0.38	4.40 ± 0.16	91.07 ± 1.00
C-2.25FMs 90 °C	50.98 ± 1.80	4.32 ± 0.30	4.69 ± 0.19	91.56 ± 0.80

## Supplementary Materials

The following are available online at https://www.mdpi.com/article/10.3390/polym13162583/s1, Figure S1: FTIR analysis of curcumin, jelly fig pectin and microcapsules, Figure S2: Thermogravimetric curves for curcumin, jelly fig pectin and microcapsules.
