# Supplementary material for: Microencapsulation of Curcumin in Crosslinked Jelly Fig Pectin Using Vacuum Spray Drying Technique for Effective Drug Delivery
Source: Polymers (Basel). 2021 Aug 4;13(16):2583. doi: 10.3390/polym13162583 (PMC8398278; doi:10.3390/polym13162583)
Supplement: Supplementary file 1 [file polymers-13-02583-s001.zip › polymers-1301801-supplementary.pdf]

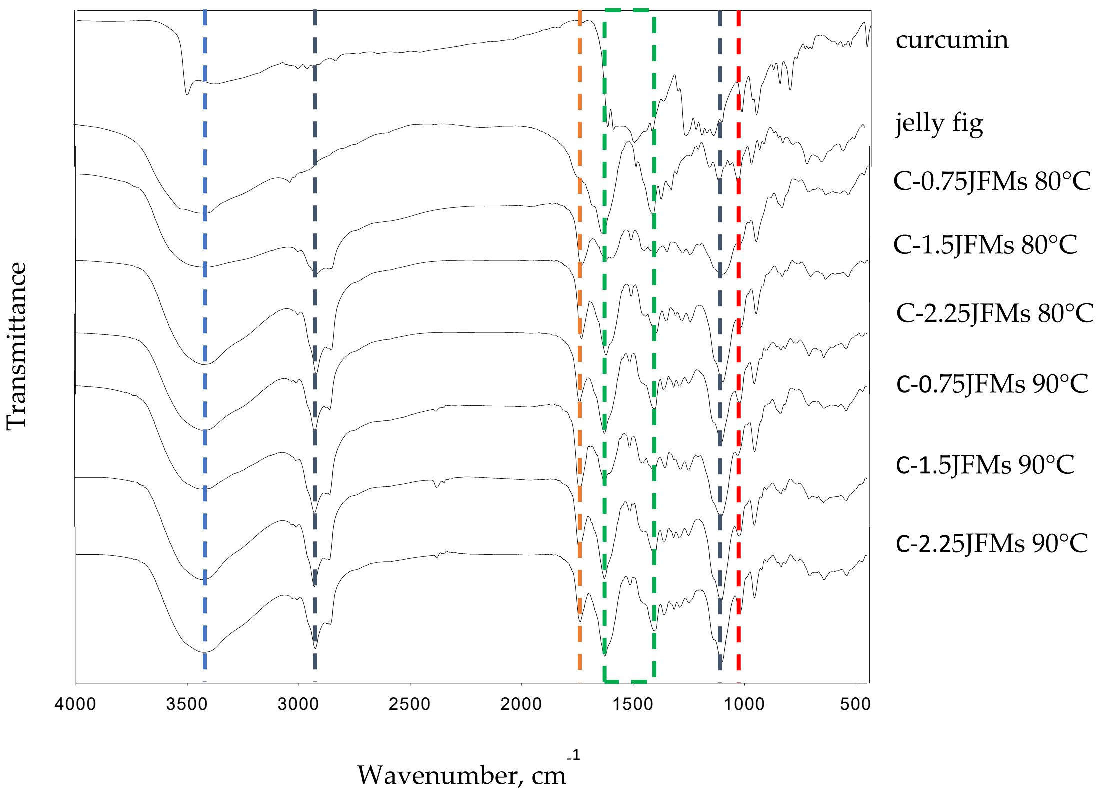

Figure S1. FTIR analysis of curcumin, jelly fig pectin and microcapsules

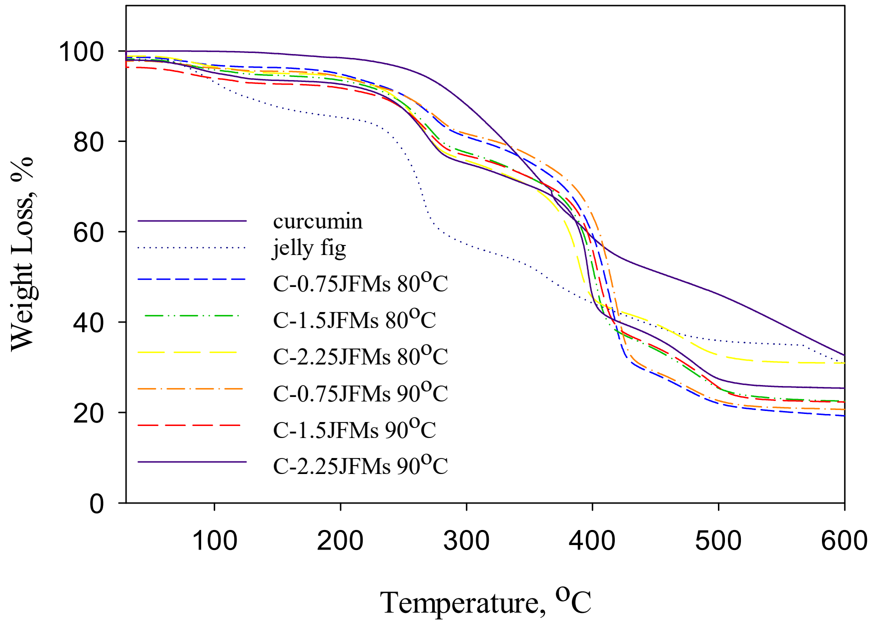

Figure S2. Thermogravimetric curves for curcumin, jelly fig and microcapsules
